# Supplementary figures and images for: S100a4+ alveolar macrophages accelerate the progression of precancerous atypical adenomatous hyperplasia by promoting the angiogenic function regulated by fatty acid metabolism
Source: eLife. 2025 Jul 14;13:RP101731. doi: 10.7554/eLife.101731 (PMC12259021; doi:10.7554/eLife.101731)

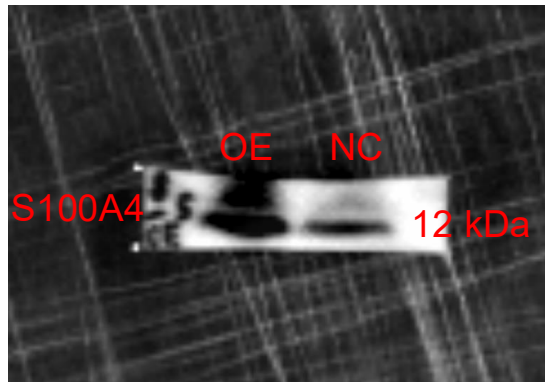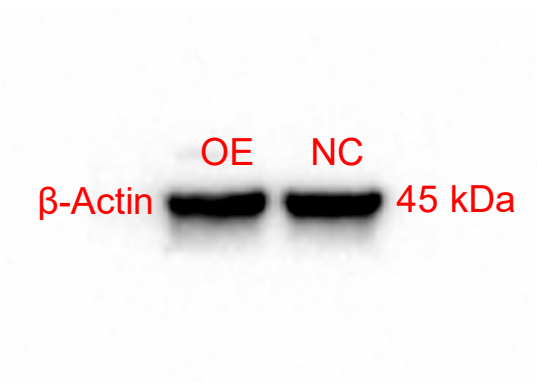

Supplement: Figure 5—source data 1. [file elife-101731-fig5-data1.zip › Figure 5-source data 1.pdf]

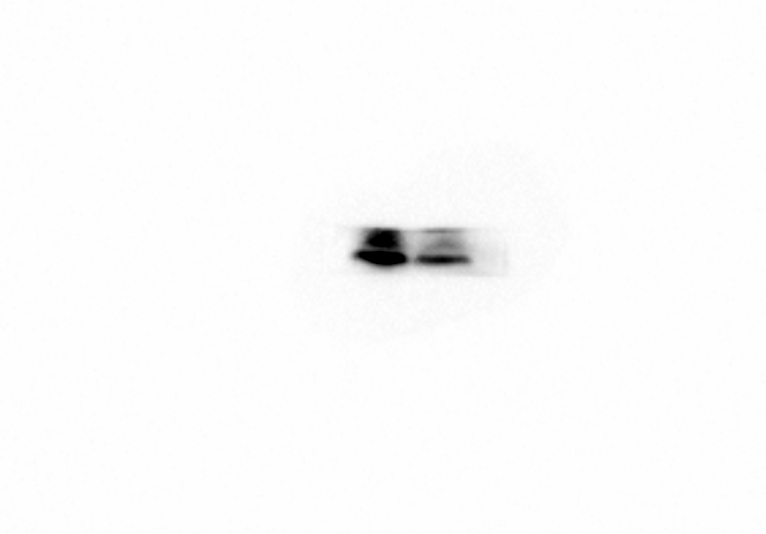

Supplement: Figure 5—source data 2. [file elife-101731-fig5-data2.zip › Figure 5-source data 2/S100A4.tif]

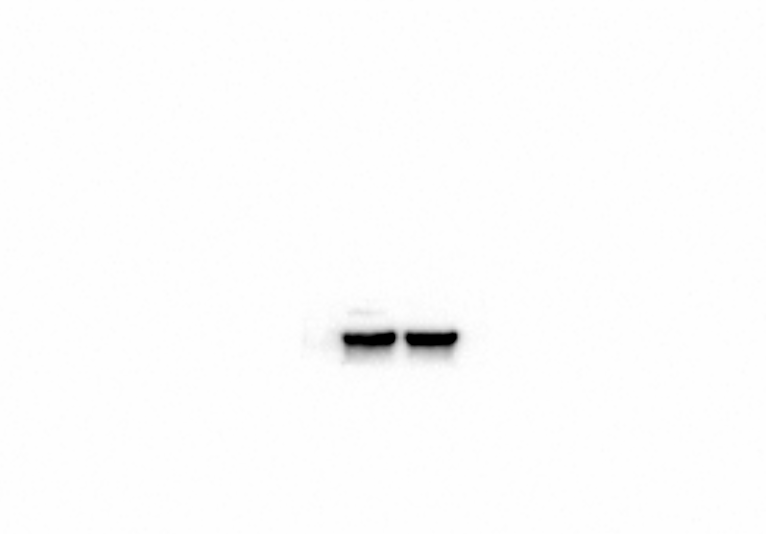

Supplement: Figure 5—source data 2. [file elife-101731-fig5-data2.zip › Figure 5-source data 2/β-Actin.tif]

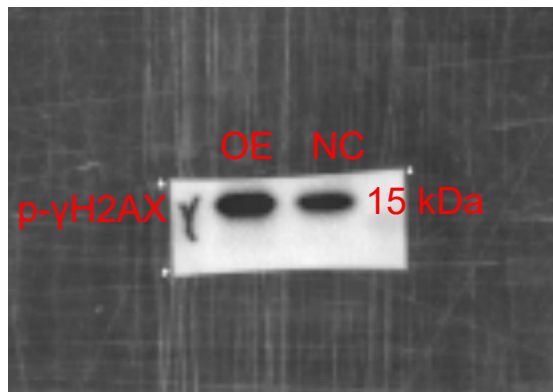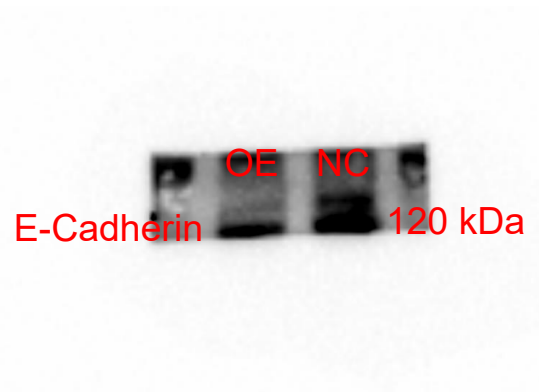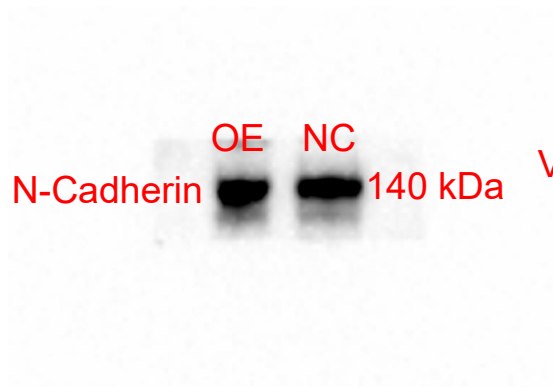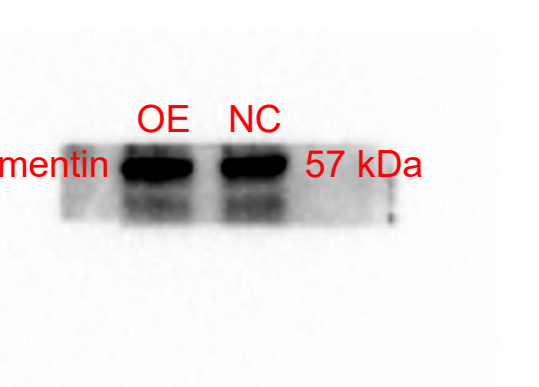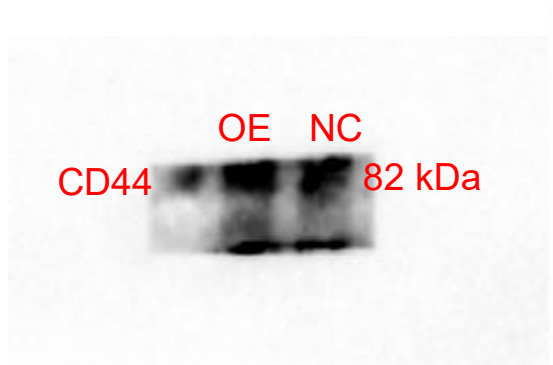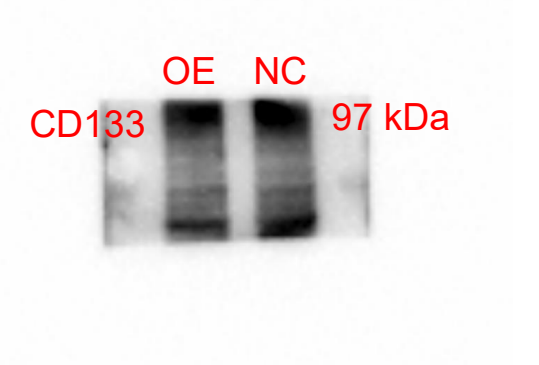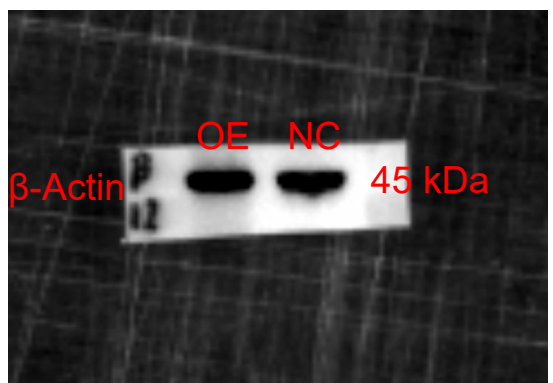

Supplement: Figure 5—source data 3. [file elife-101731-fig5-data3.zip › Figure 5-source data 3.pdf]

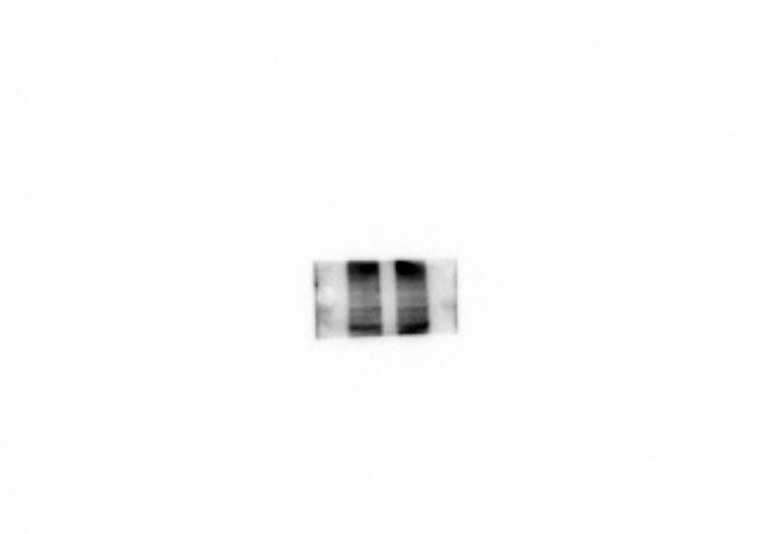

Supplement: Figure 5—source data 4. [file elife-101731-fig5-data4.zip › Figure 5-source data 4/CD133.tif]

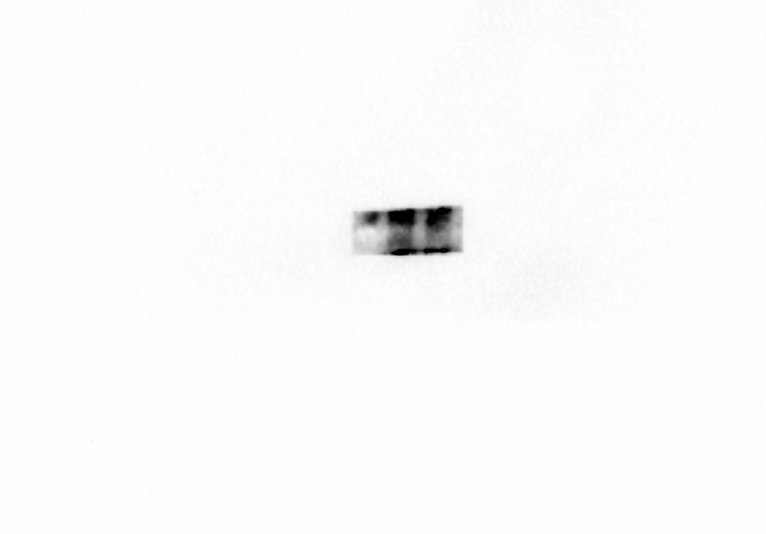

Supplement: Figure 5—source data 4. [file elife-101731-fig5-data4.zip › Figure 5-source data 4/CD44.tif]

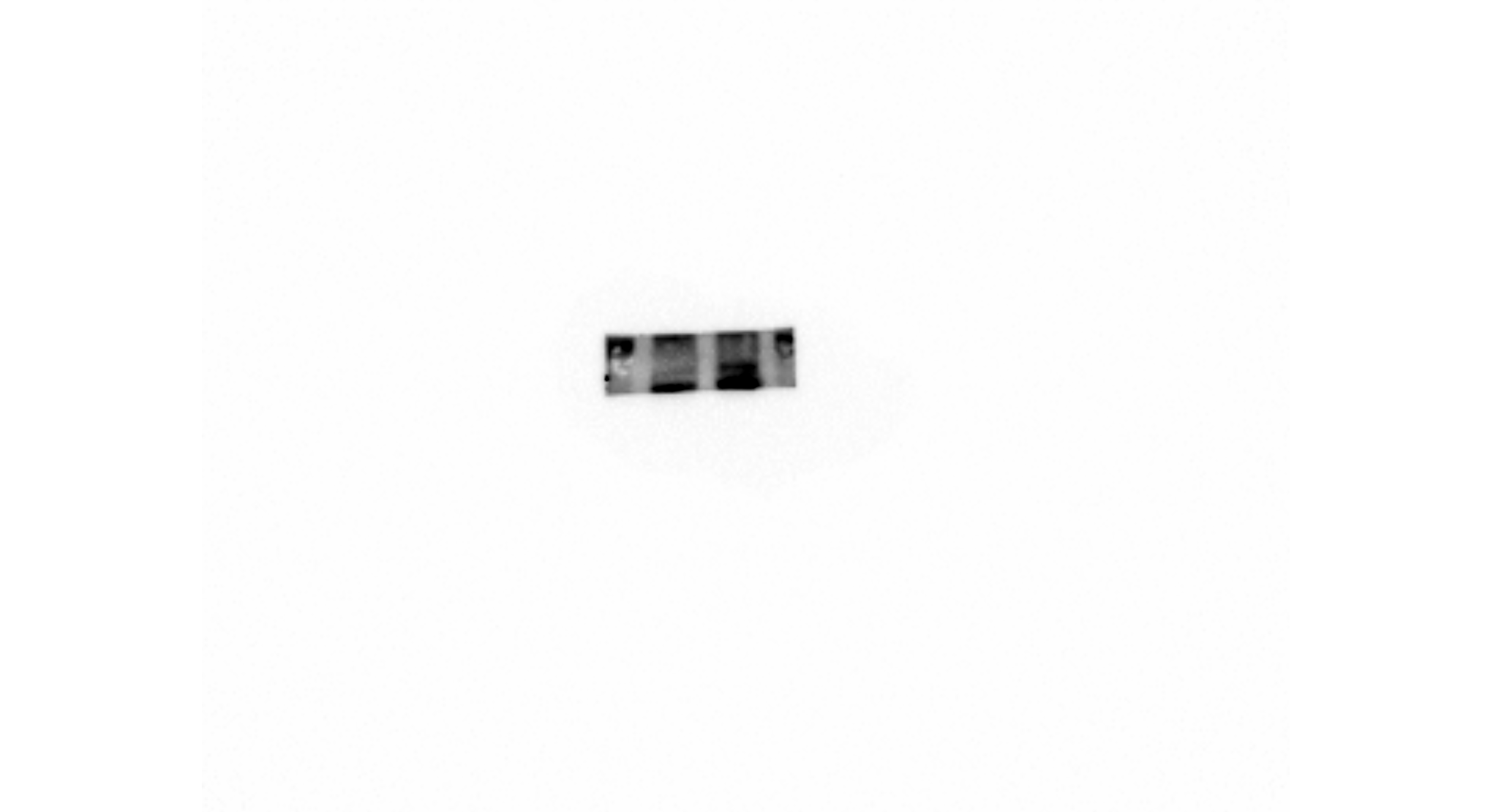

Supplement: Figure 5—source data 4. [file elife-101731-fig5-data4.zip › Figure 5-source data 4/E-Cadherin.tif]

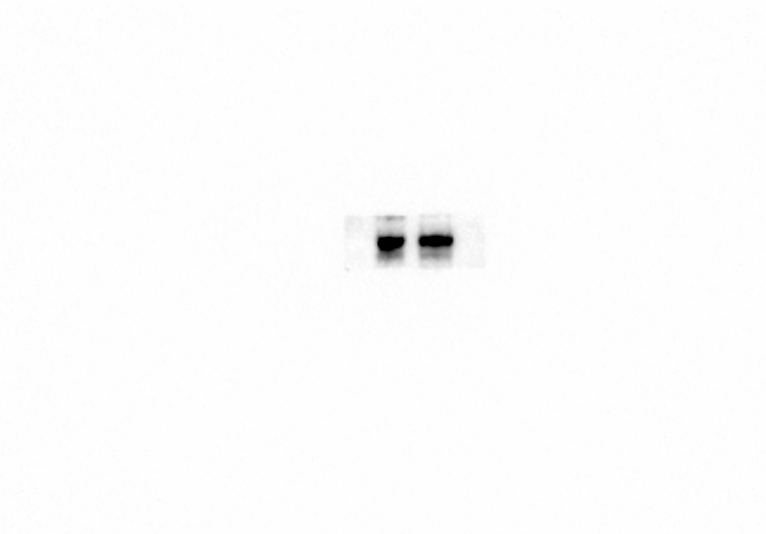

Supplement: Figure 5—source data 4. [file elife-101731-fig5-data4.zip › Figure 5-source data 4/N-Cadherin.tif]

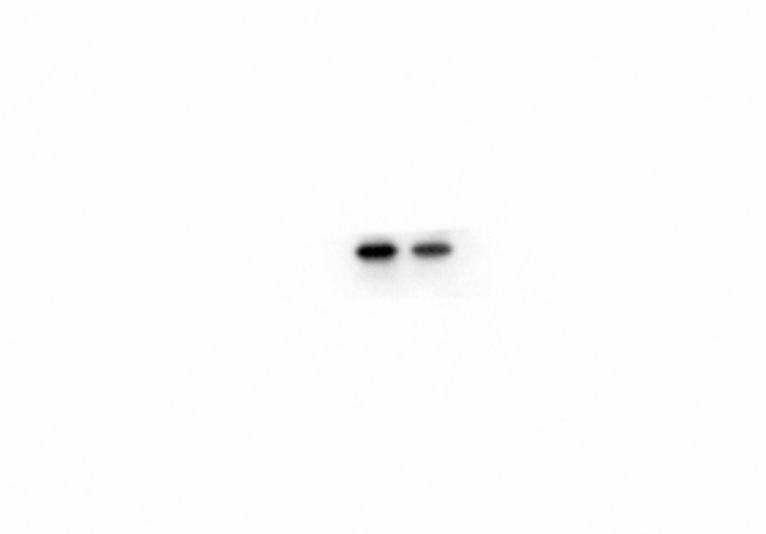

Supplement: Figure 5—source data 4. [file elife-101731-fig5-data4.zip › Figure 5-source data 4/p-γH2AX.tif]

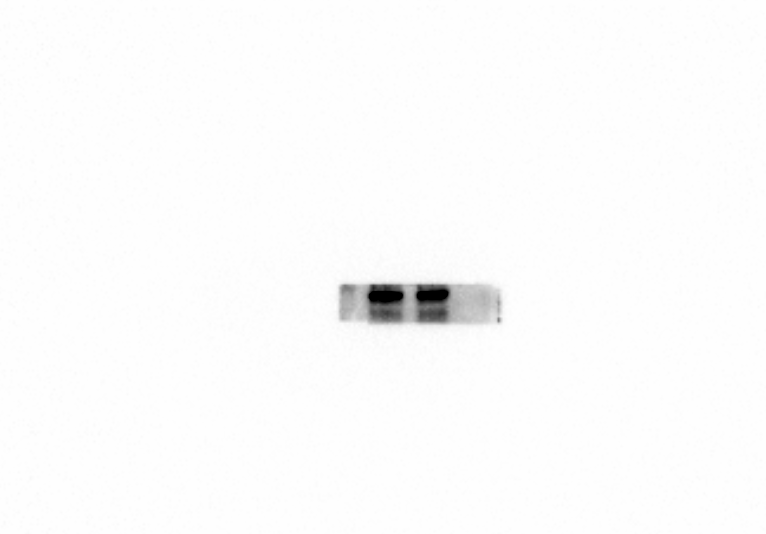

Supplement: Figure 5—source data 4. [file elife-101731-fig5-data4.zip › Figure 5-source data 4/Vimentin.tif]

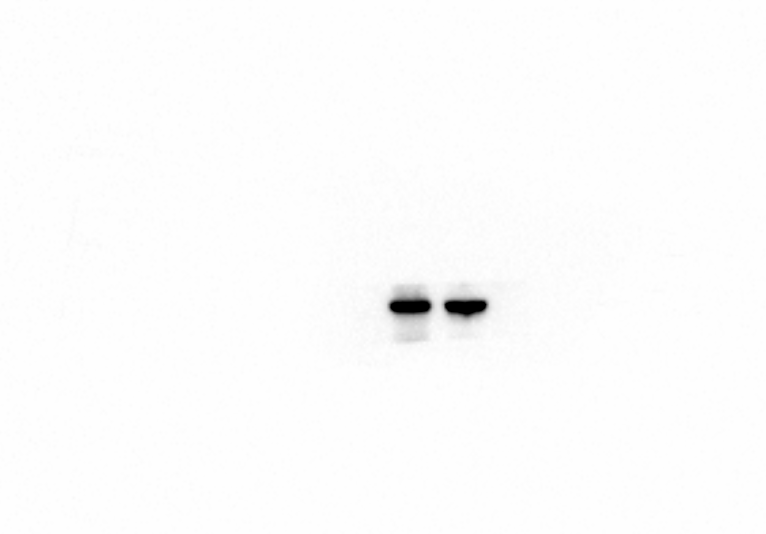

Supplement: Figure 5—source data 4. [file elife-101731-fig5-data4.zip › Figure 5-source data 4/β-Actin.tif]

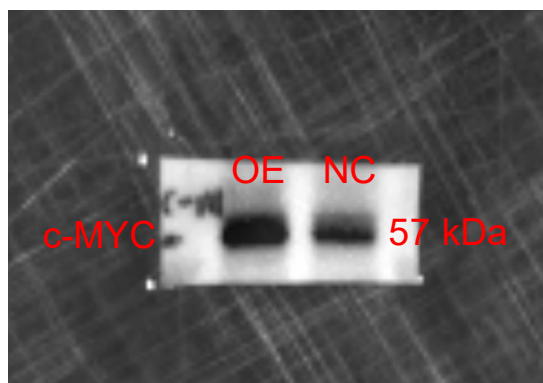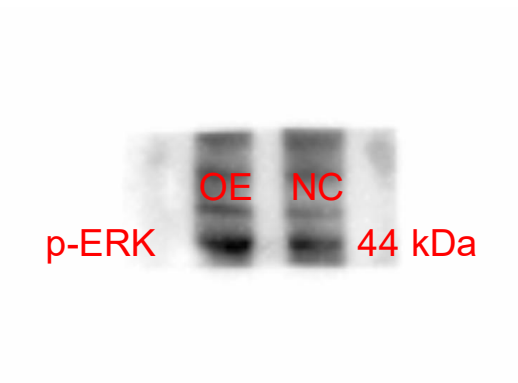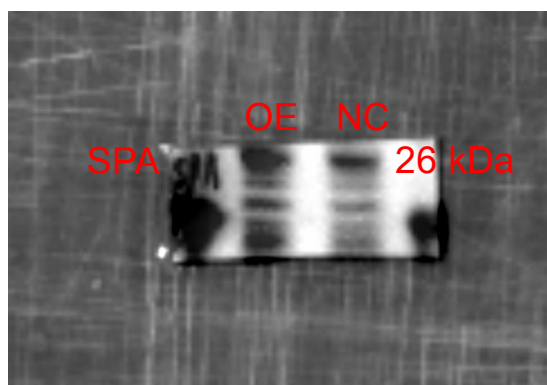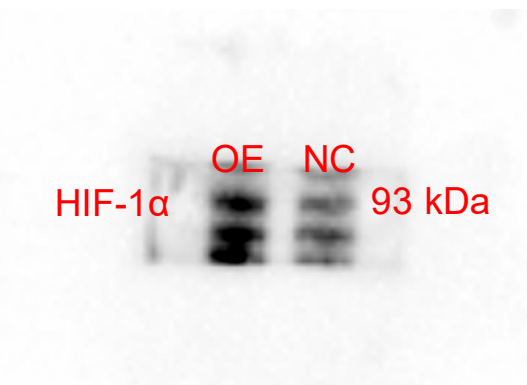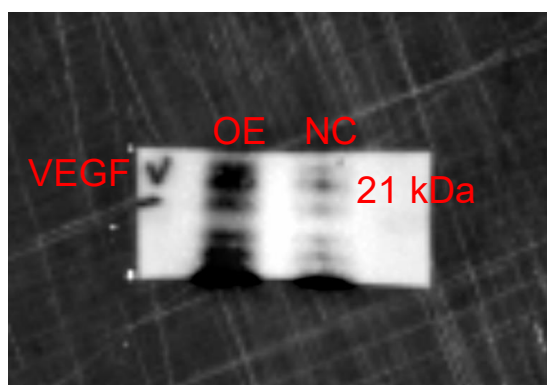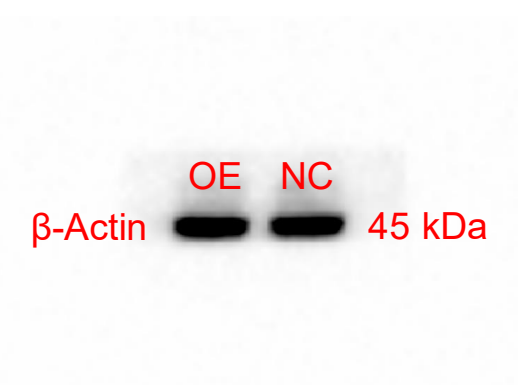

Supplement: Figure 5—source data 5. [file elife-101731-fig5-data5.zip › Figure 5-source data 5.pdf]

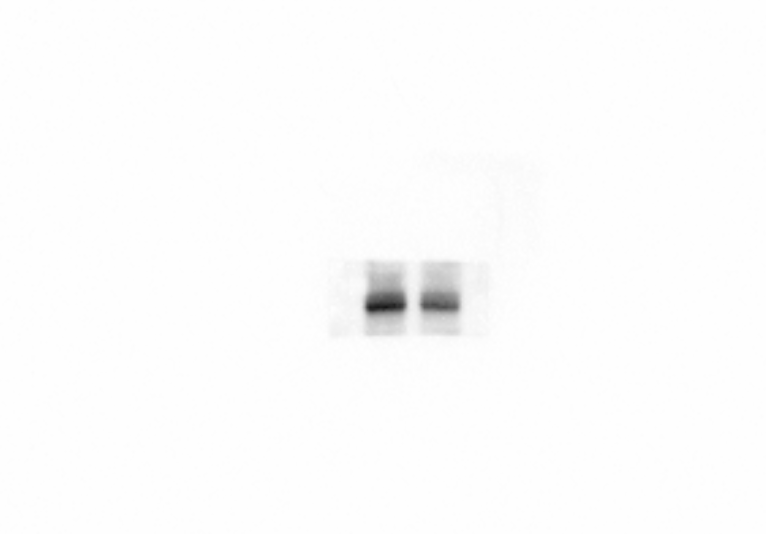

Supplement: Figure 5—source data 6. [file elife-101731-fig5-data6.zip › Figure 5-source data 6/c-MYC.tif]

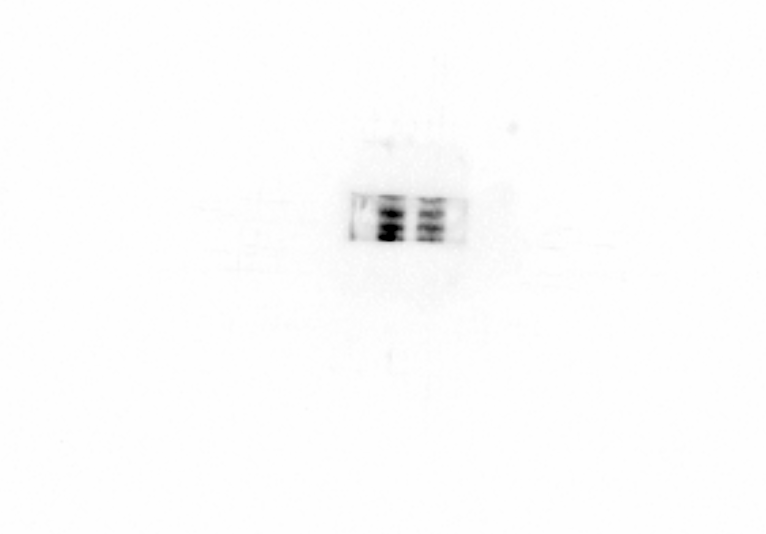

Supplement: Figure 5—source data 6. [file elife-101731-fig5-data6.zip › Figure 5-source data 6/HIF-1α.tif]

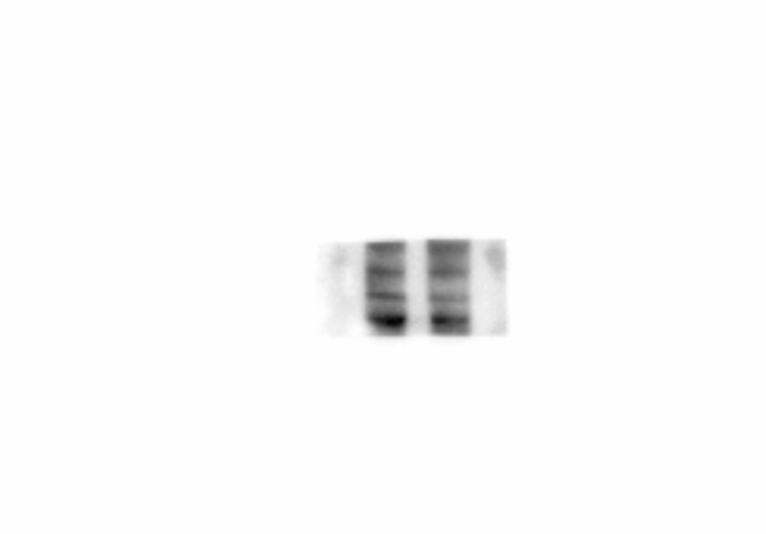

Supplement: Figure 5—source data 6. [file elife-101731-fig5-data6.zip › Figure 5-source data 6/p-ERK.tif]

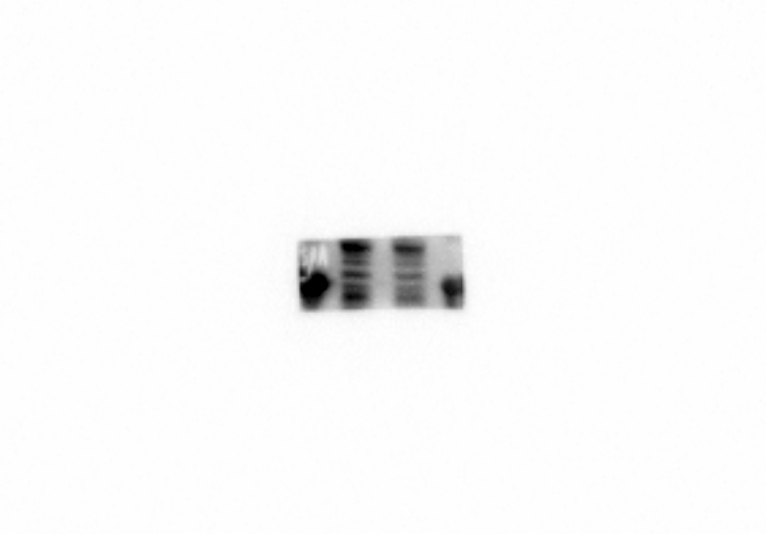

Supplement: Figure 5—source data 6. [file elife-101731-fig5-data6.zip › Figure 5-source data 6/SPA.tif]

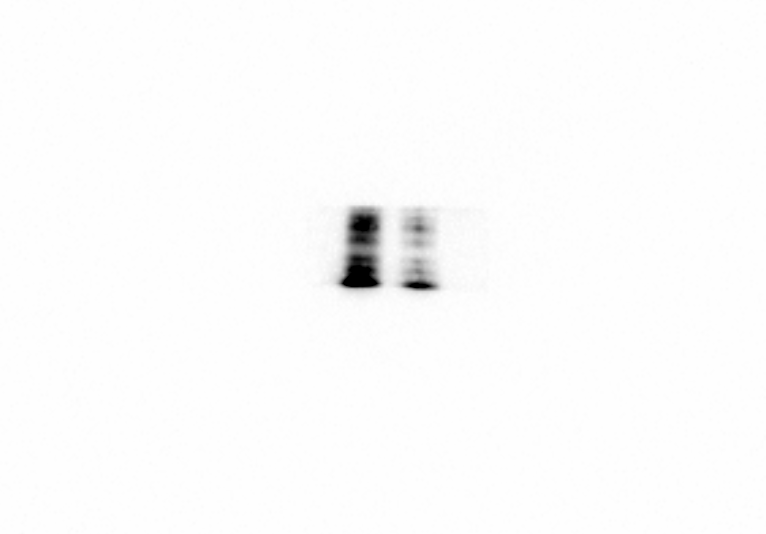

Supplement: Figure 5—source data 6. [file elife-101731-fig5-data6.zip › Figure 5-source data 6/VEGF.tif]

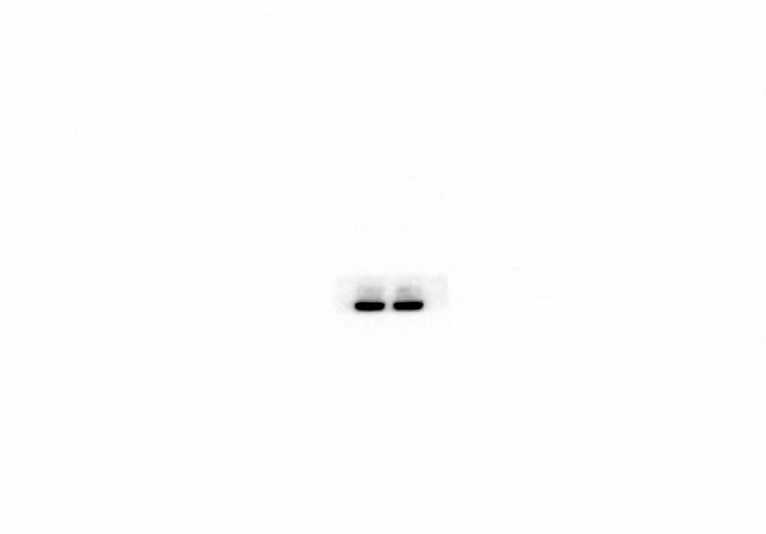

Supplement: Figure 5—source data 6. [file elife-101731-fig5-data6.zip › Figure 5-source data 6/β-Actin.tif]

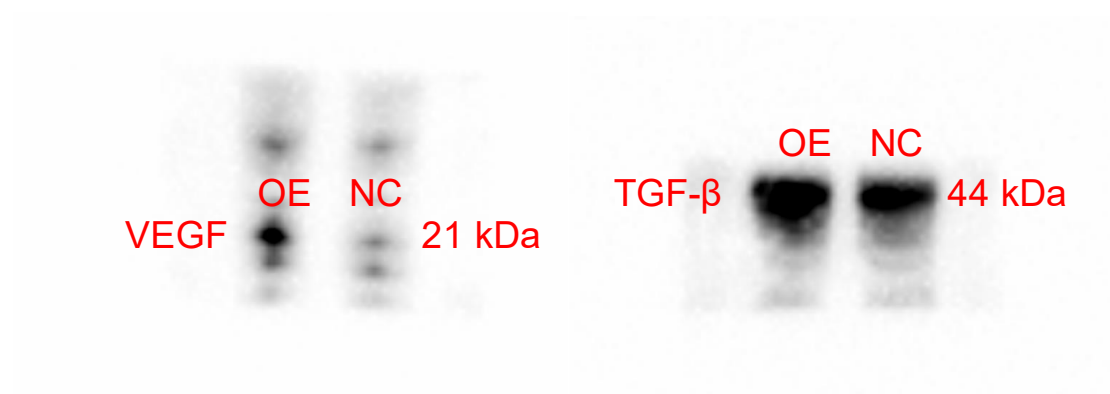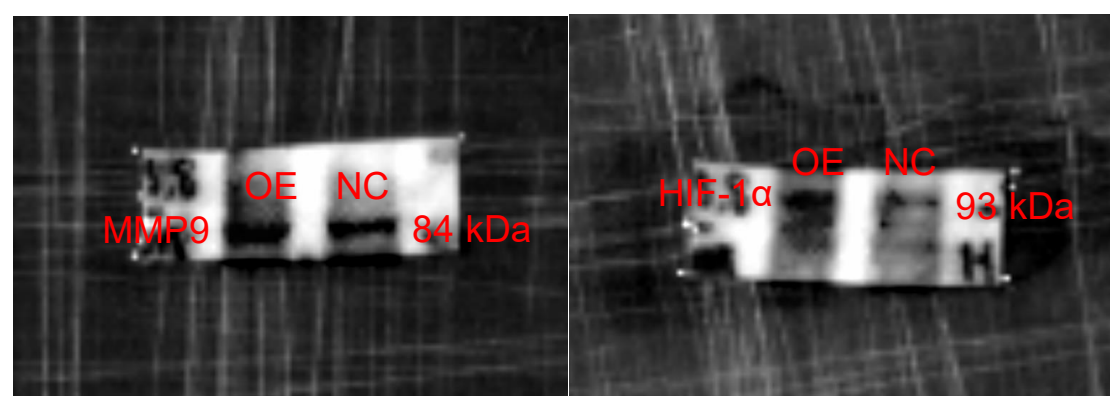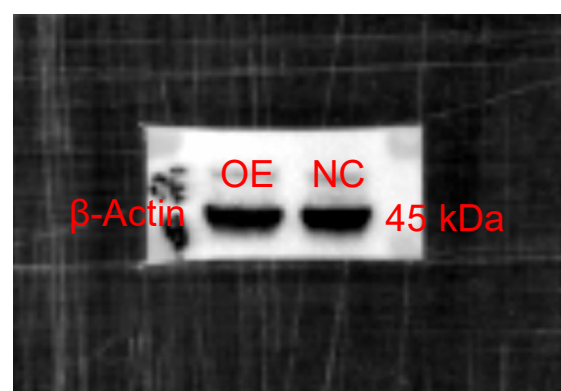

Supplement: Figure 5—source data 7. [file elife-101731-fig5-data7.zip › Figure 5-source data 7.pdf]

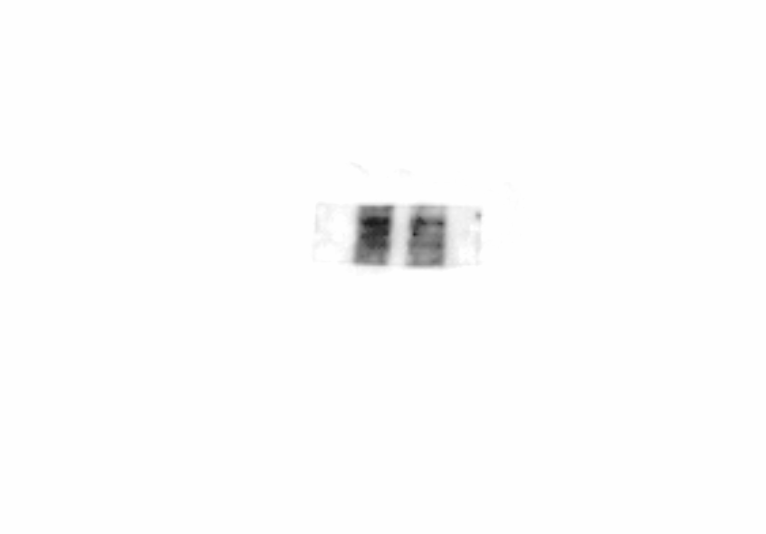

Supplement: Figure 5—source data 8. [file elife-101731-fig5-data8.zip › Figure 5-source data 8/HIF-1α.tif]

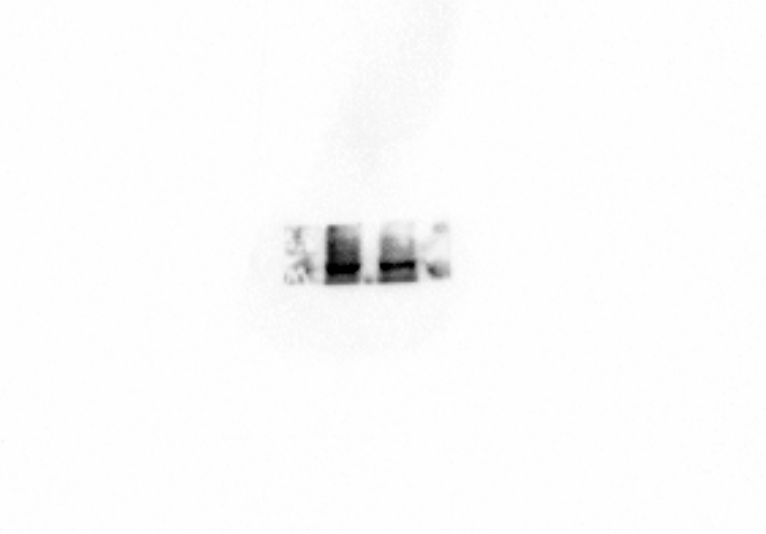

Supplement: Figure 5—source data 8. [file elife-101731-fig5-data8.zip › Figure 5-source data 8/MMP9.tif]

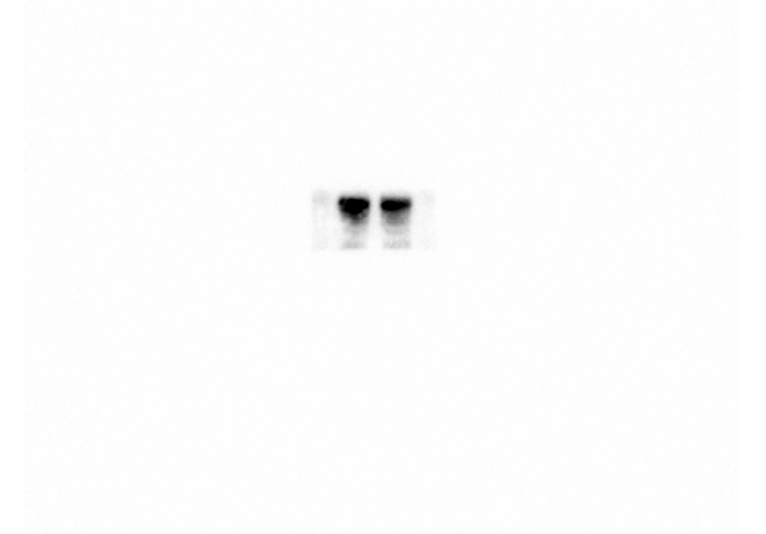

Supplement: Figure 5—source data 8. [file elife-101731-fig5-data8.zip › Figure 5-source data 8/TGF-β.tif]

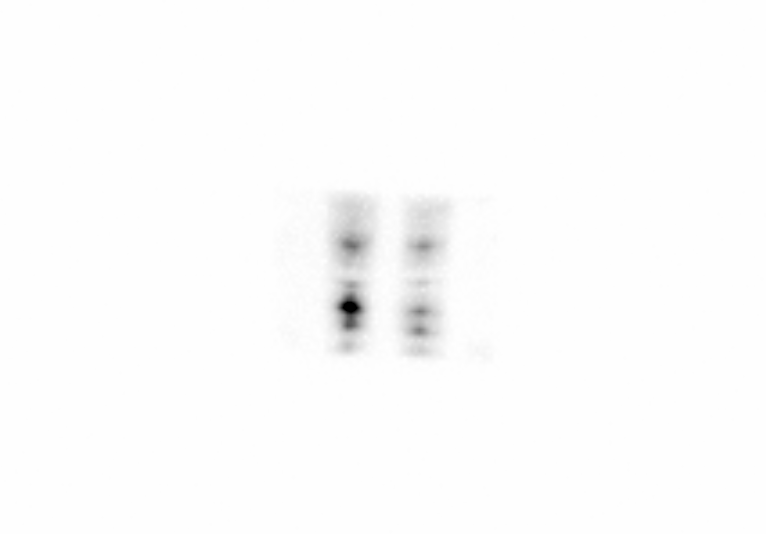

Supplement: Figure 5—source data 8. [file elife-101731-fig5-data8.zip › Figure 5-source data 8/VEGF.tif]

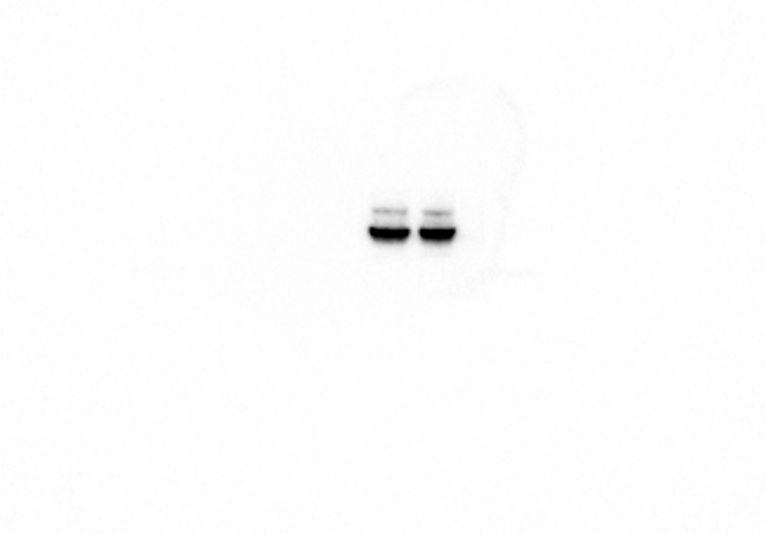

Supplement: Figure 5—source data 8. [file elife-101731-fig5-data8.zip › Figure 5-source data 8/β-Actin.tif]

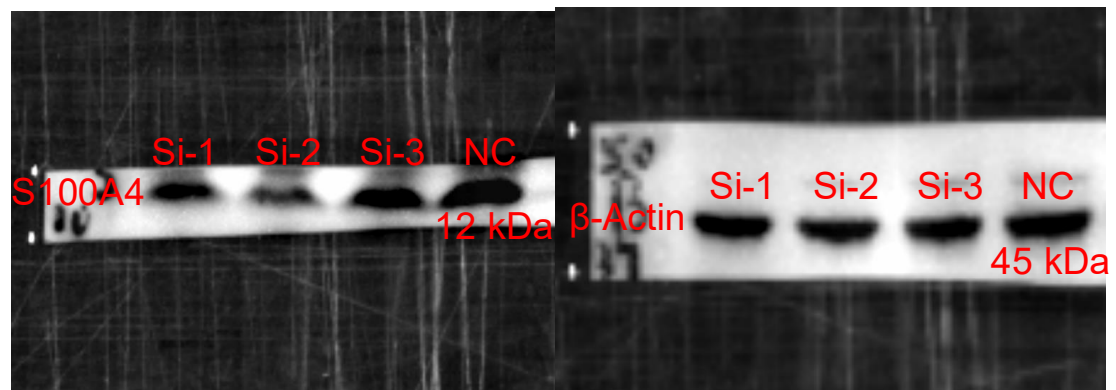

Supplement: Figure 5—figure supplement 1—source data 1. [file elife-101731-fig5-figsupp1-data1.zip › Figure 5-figure supplement 1-source data 1.pdf]

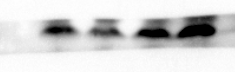

Supplement: Figure 5—figure supplement 1—source data 2. [file elife-101731-fig5-figsupp1-data2.zip › Figure 5-figure supplement 1-source data 2/S100A4.tif]

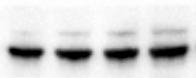

Supplement: Figure 5—figure supplement 1—source data 2. [file elife-101731-fig5-figsupp1-data2.zip › Figure 5-figure supplement 1-source data 2/β-Actin.tif]

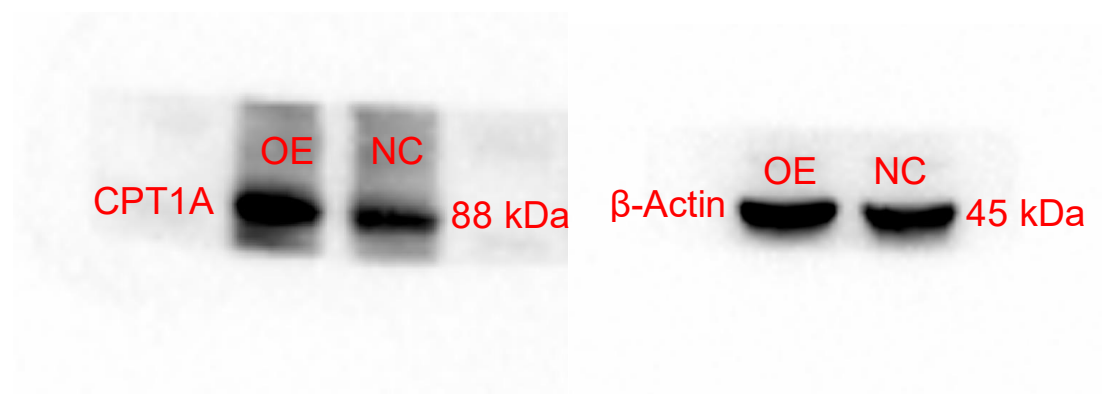

Supplement: Figure 6—source data 1. [file elife-101731-fig6-data1.zip › Figure 6-source data 1.pdf]

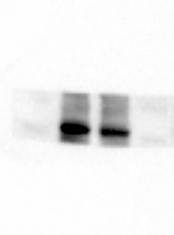

Supplement: Figure 6—source data 2. [file elife-101731-fig6-data2.zip › Figure 6-source data 2/CPT1A.tif]

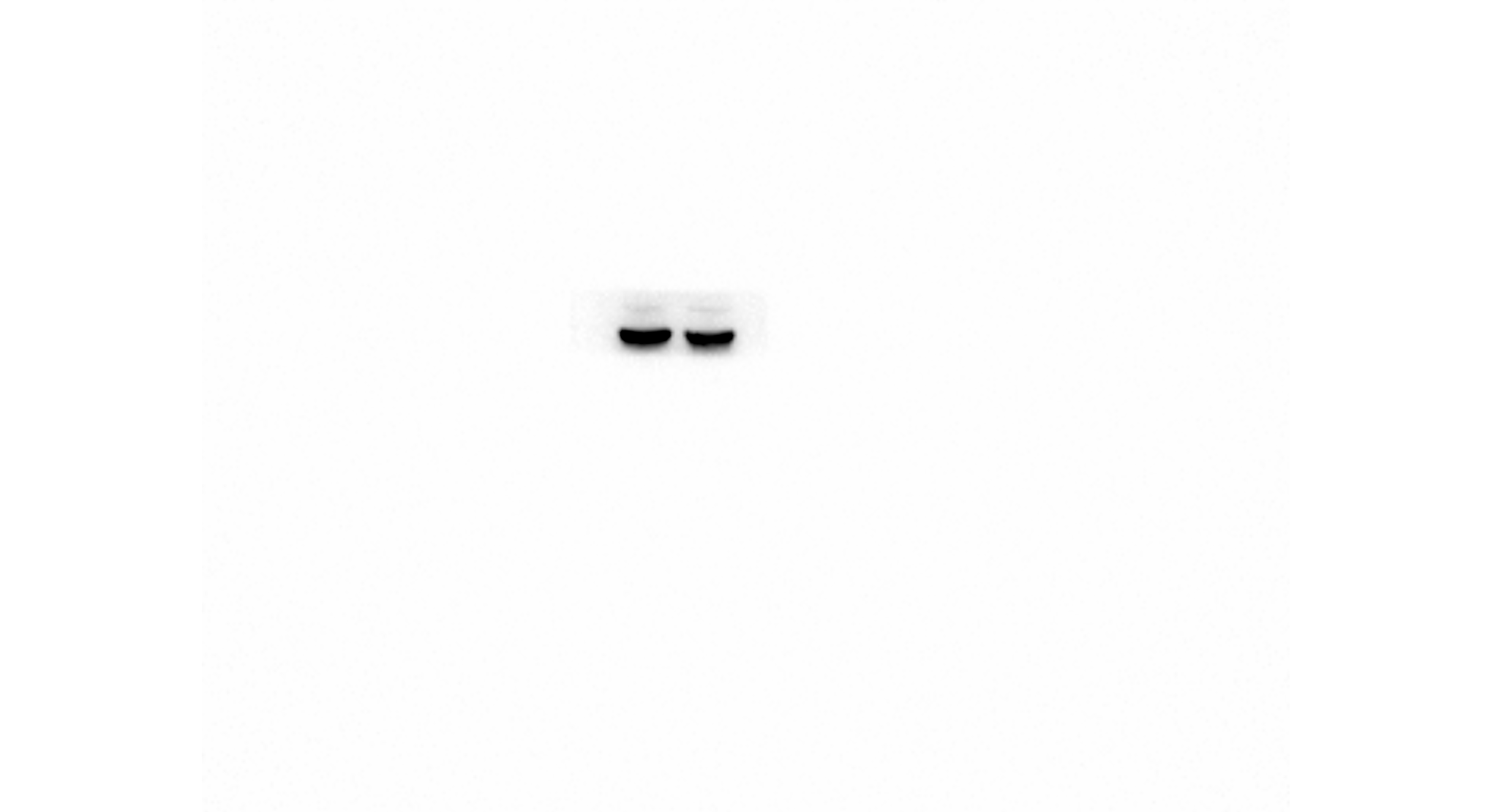

Supplement: Figure 6—source data 2. [file elife-101731-fig6-data2.zip › Figure 6-source data 2/β-Actin.tif]

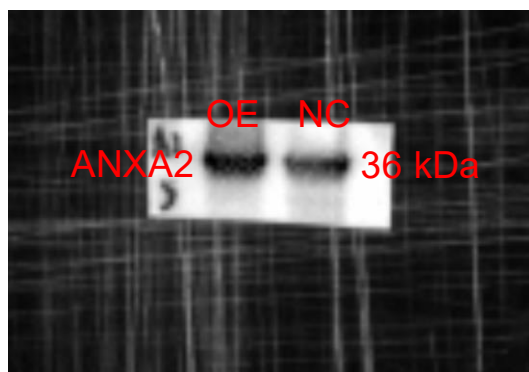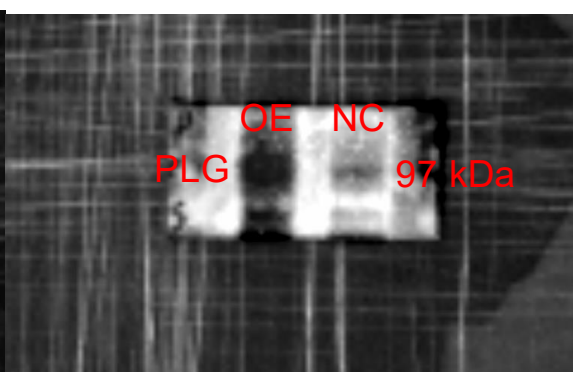

MH-S

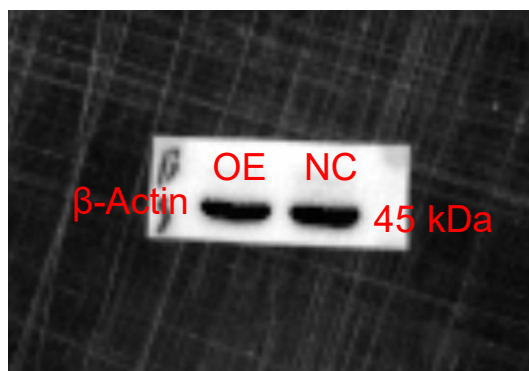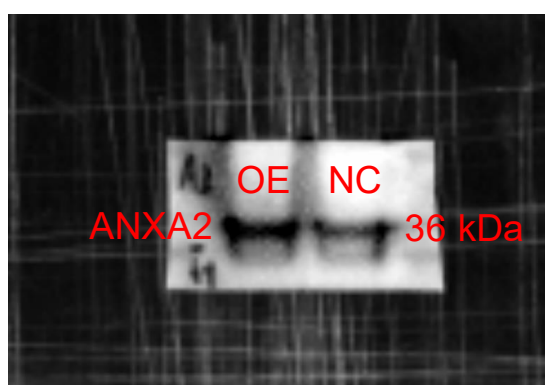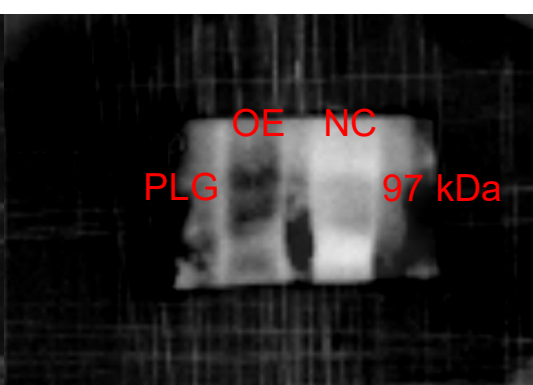

HUVEC

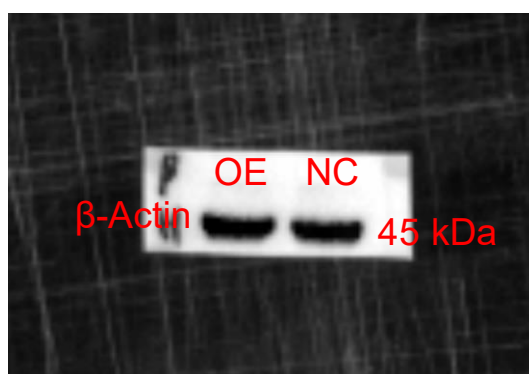

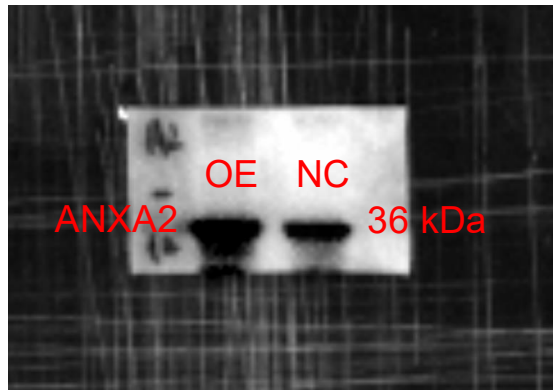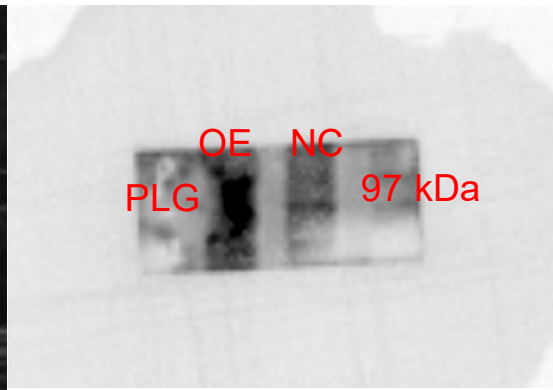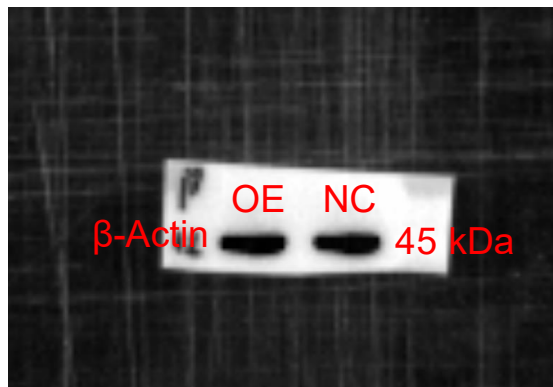

MLE12

Supplement: Figure 6—source data 3. [file elife-101731-fig6-data3.zip › Figure 6-source data 3.pdf]

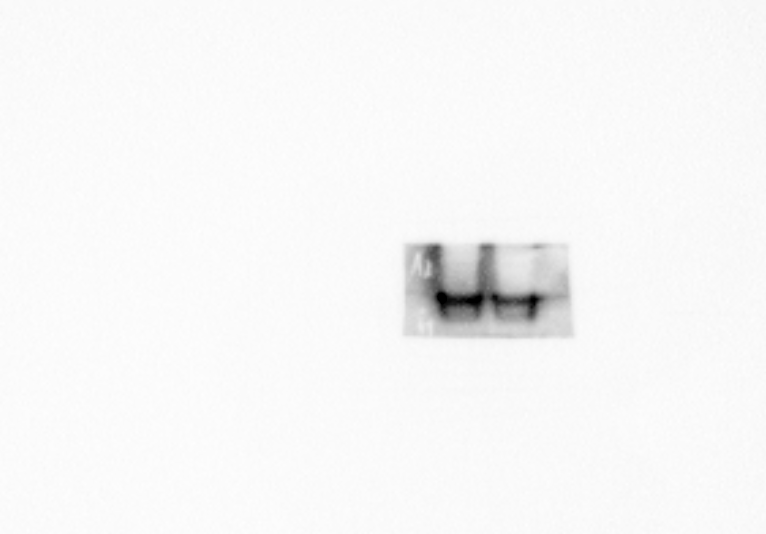

Supplement: Figure 6—source data 4. [file elife-101731-fig6-data4.zip › Figure 6-source data 4/ANXA2-HUVEC.tif]

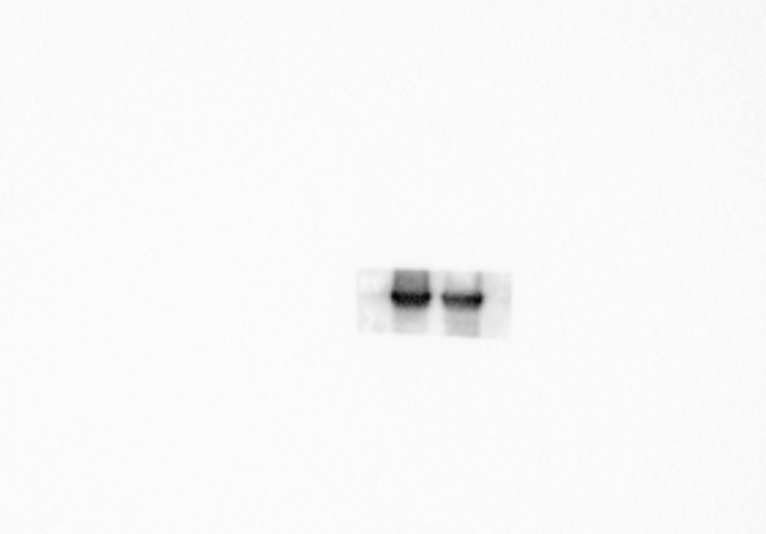

Supplement: Figure 6—source data 4. [file elife-101731-fig6-data4.zip › Figure 6-source data 4/ANXA2-MH-S.tif]

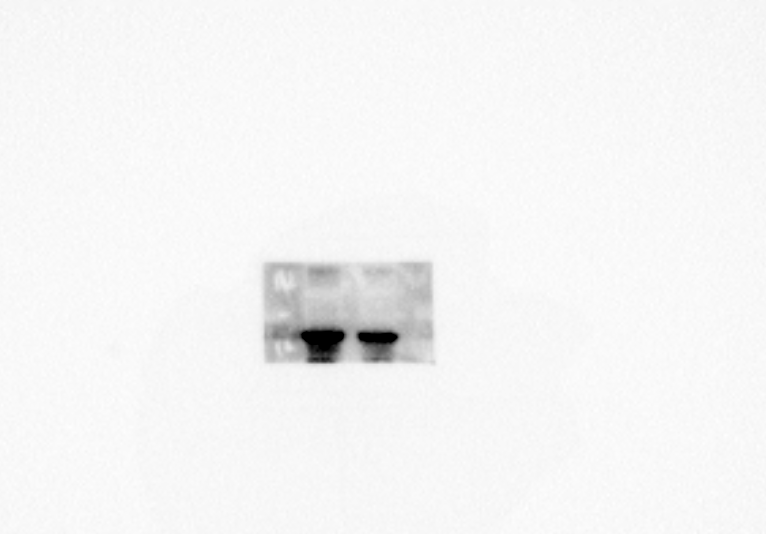

Supplement: Figure 6—source data 4. [file elife-101731-fig6-data4.zip › Figure 6-source data 4/ANXA2-MLE12.tif]

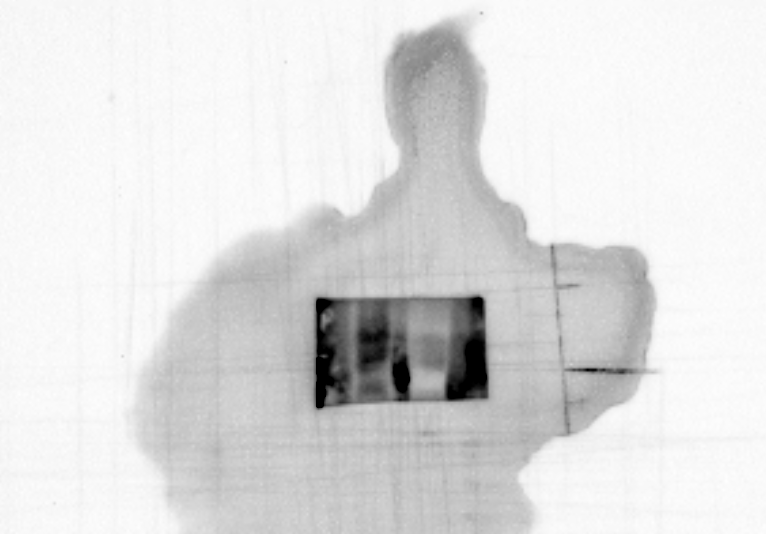

Supplement: Figure 6—source data 4. [file elife-101731-fig6-data4.zip › Figure 6-source data 4/PLG-HUVEC.tif]

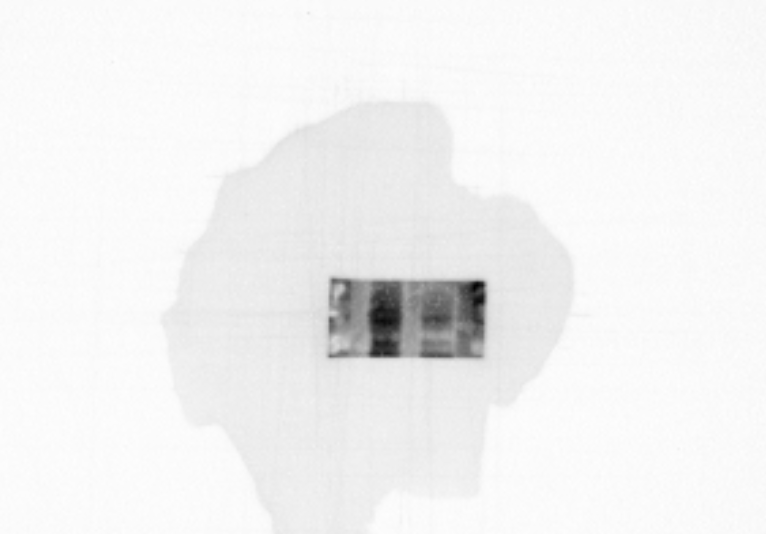

Supplement: Figure 6—source data 4. [file elife-101731-fig6-data4.zip › Figure 6-source data 4/PLG-MH-S.tif]

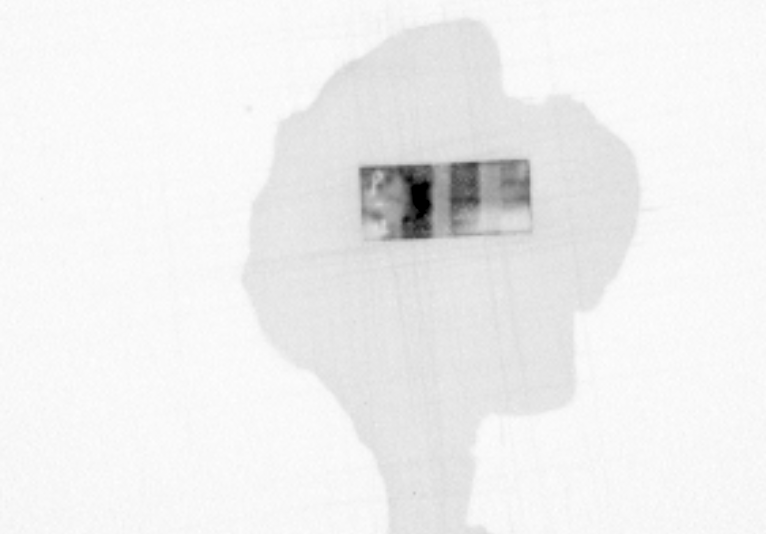

Supplement: Figure 6—source data 4. [file elife-101731-fig6-data4.zip › Figure 6-source data 4/PLG-MLE12.tif]

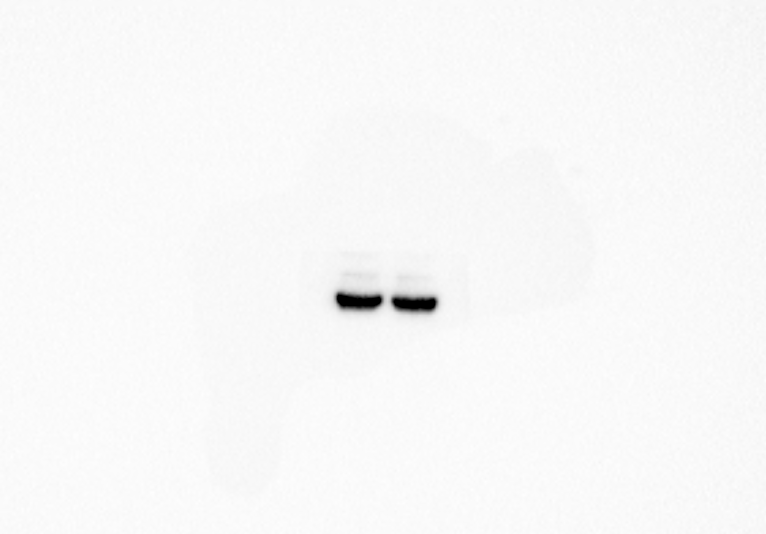

Supplement: Figure 6—source data 4. [file elife-101731-fig6-data4.zip › Figure 6-source data 4/β-Actin-HUVEC.tif]

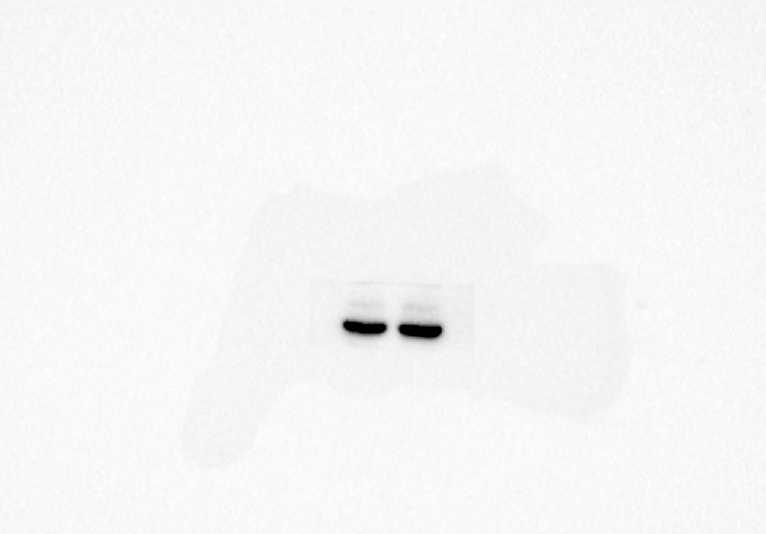

Supplement: Figure 6—source data 4. [file elife-101731-fig6-data4.zip › Figure 6-source data 4/β-Actin-MH-S.tif]

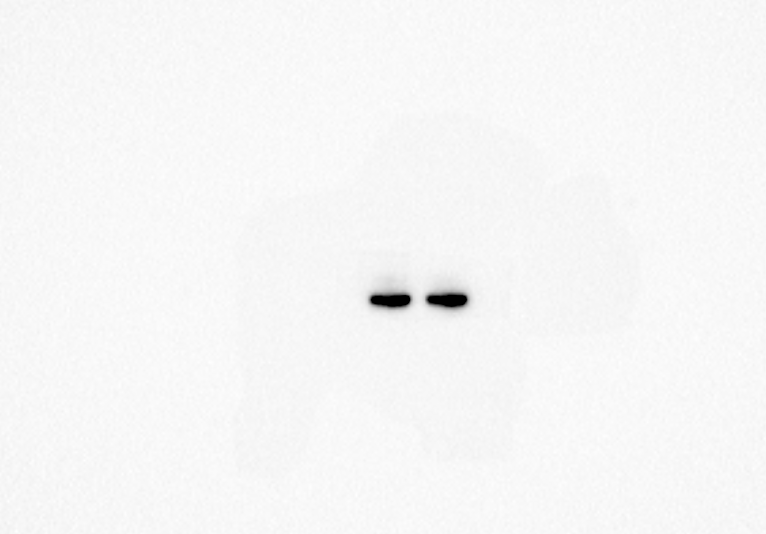

Supplement: Figure 6—source data 4. [file elife-101731-fig6-data4.zip › Figure 6-source data 4/β-Actin-MLE12.tif]

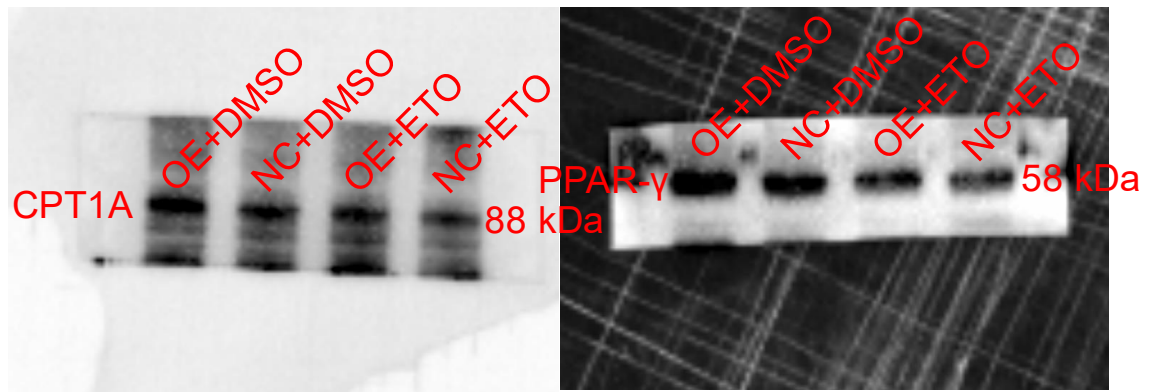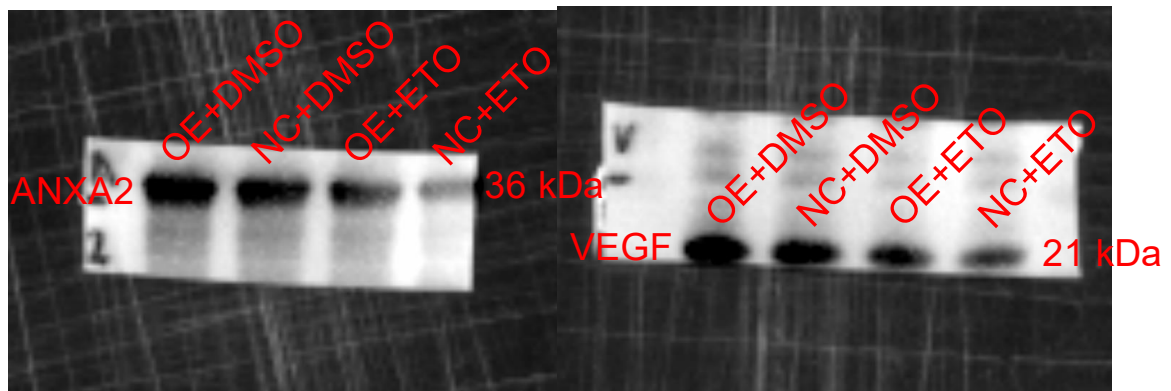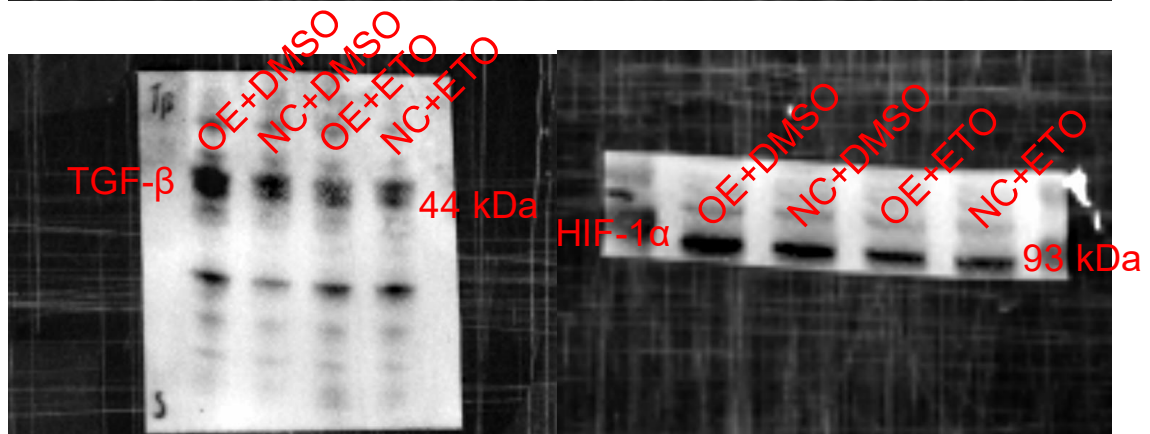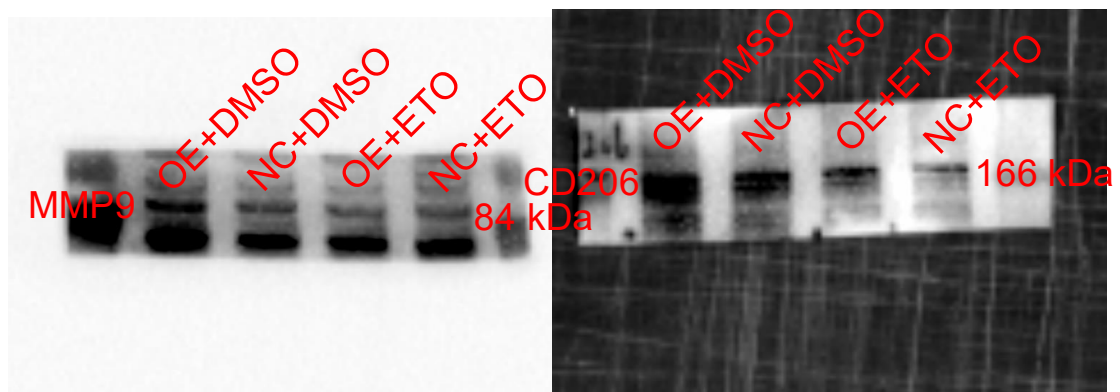

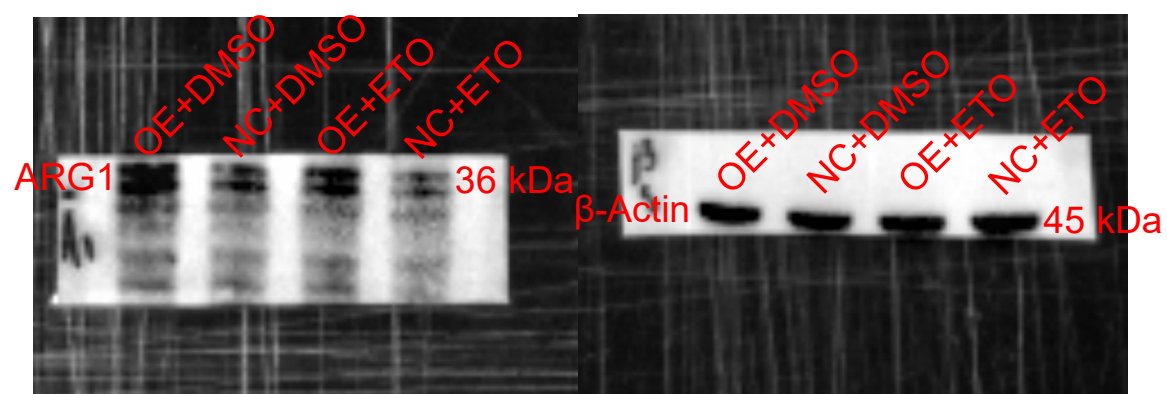

Supplement: Figure 6—source data 5. [file elife-101731-fig6-data5.zip › Figure 6-source data 5.pdf]

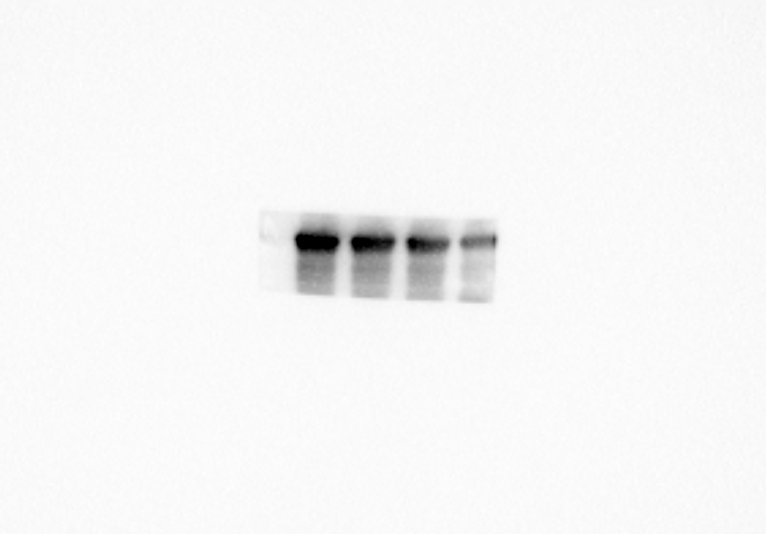

Supplement: Figure 6—source data 6. [file elife-101731-fig6-data6.zip › Figure 6-source data 6/ANXA2.tif]

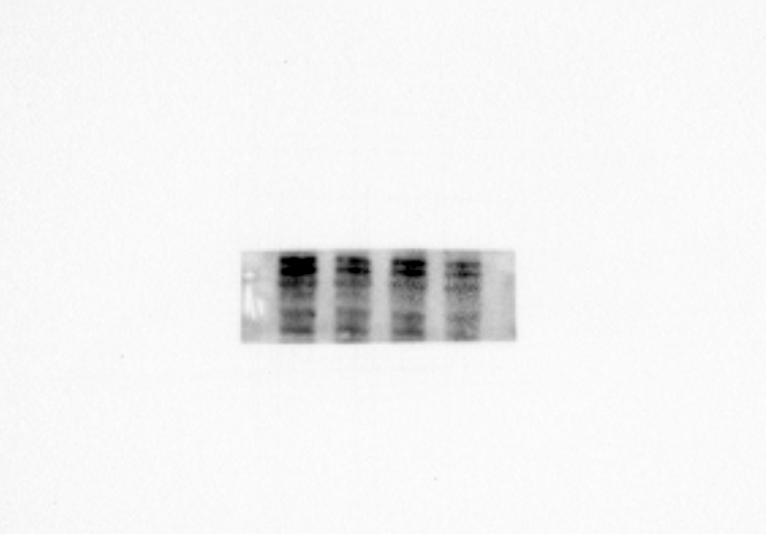

Supplement: Figure 6—source data 6. [file elife-101731-fig6-data6.zip › Figure 6-source data 6/ARG1.tif]

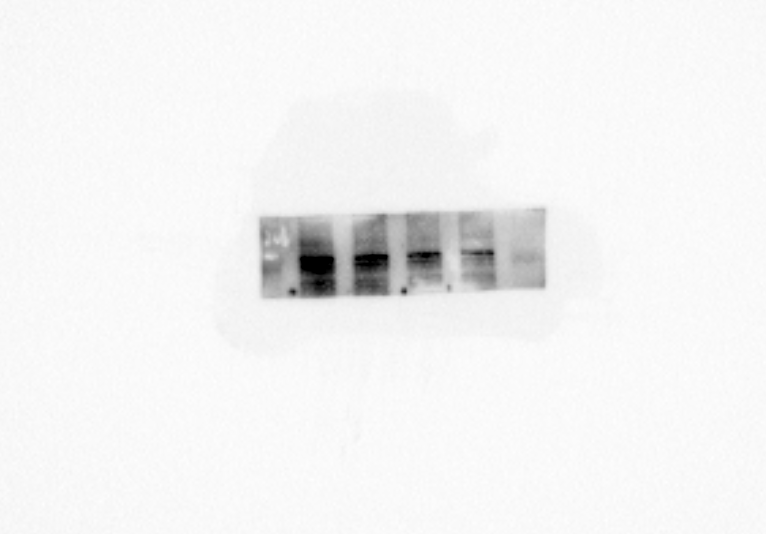

Supplement: Figure 6—source data 6. [file elife-101731-fig6-data6.zip › Figure 6-source data 6/CD206.tif]

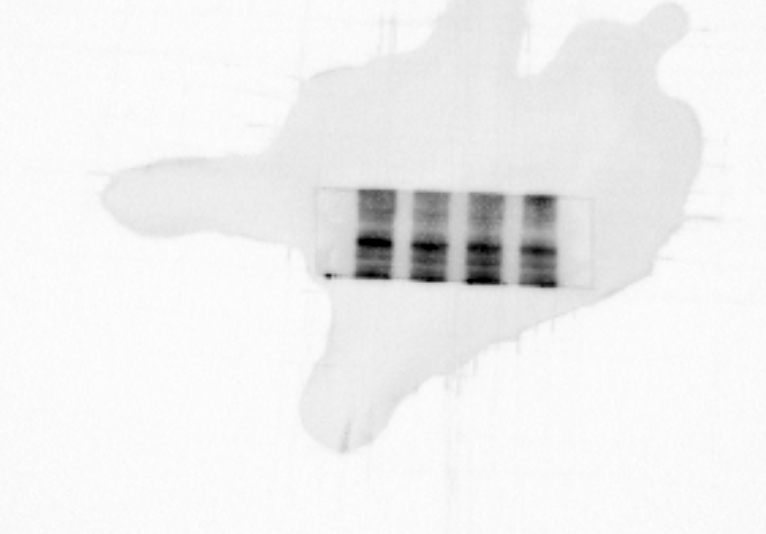

Supplement: Figure 6—source data 6. [file elife-101731-fig6-data6.zip › Figure 6-source data 6/CPT1A.tif]

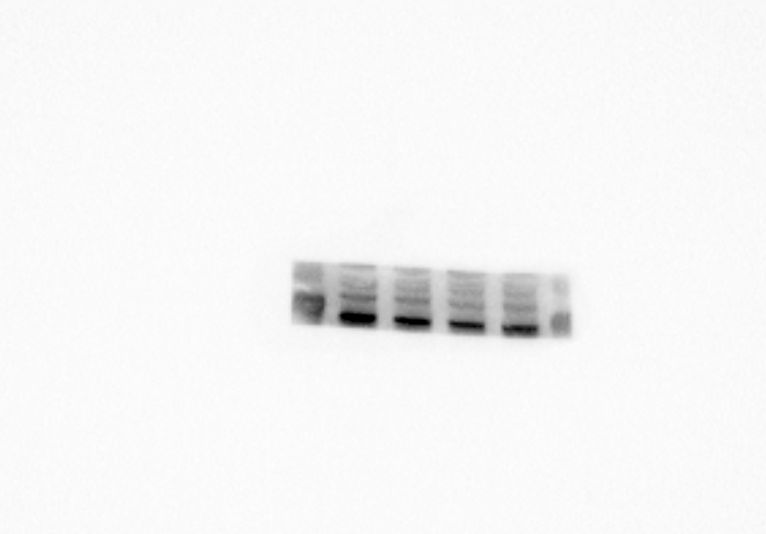

Supplement: Figure 6—source data 6. [file elife-101731-fig6-data6.zip › Figure 6-source data 6/HIF-1α.tif]

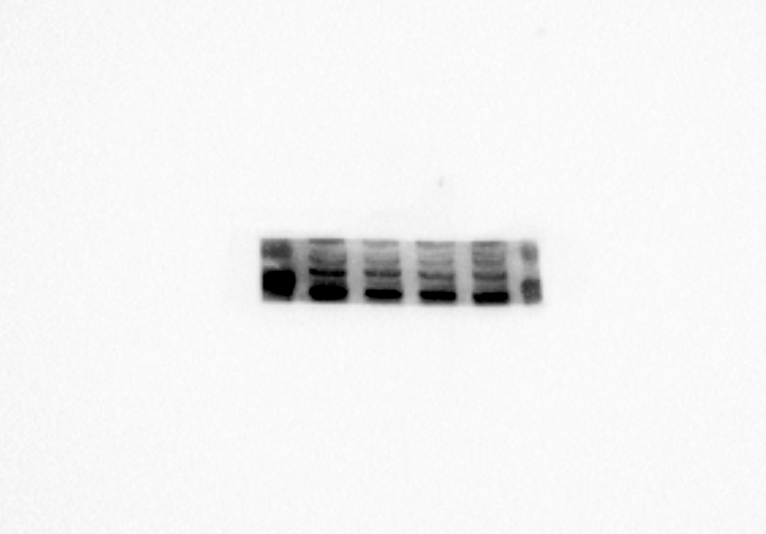

Supplement: Figure 6—source data 6. [file elife-101731-fig6-data6.zip › Figure 6-source data 6/MMP9.tif]

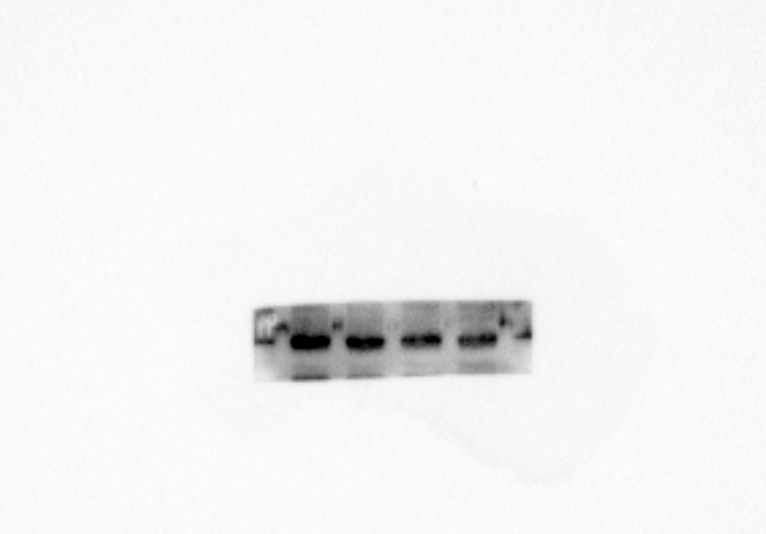

Supplement: Figure 6—source data 6. [file elife-101731-fig6-data6.zip › Figure 6-source data 6/PPAR-γ.tif]

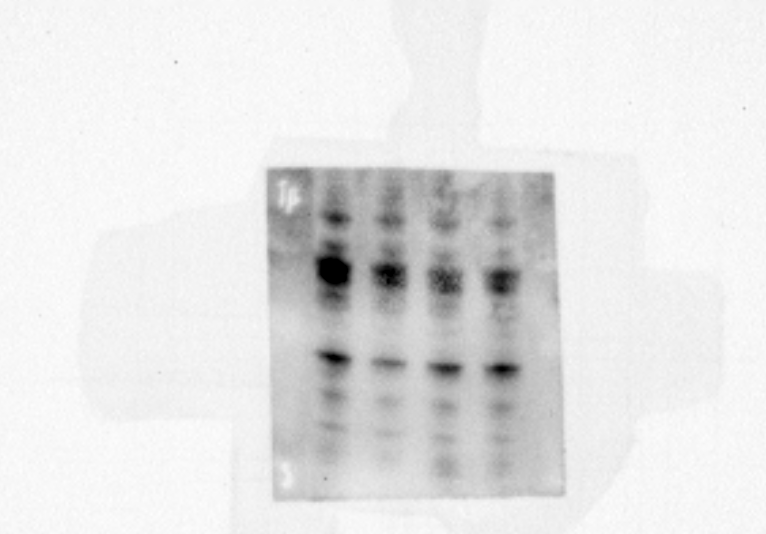

Supplement: Figure 6—source data 6. [file elife-101731-fig6-data6.zip › Figure 6-source data 6/TGF-β.tif]

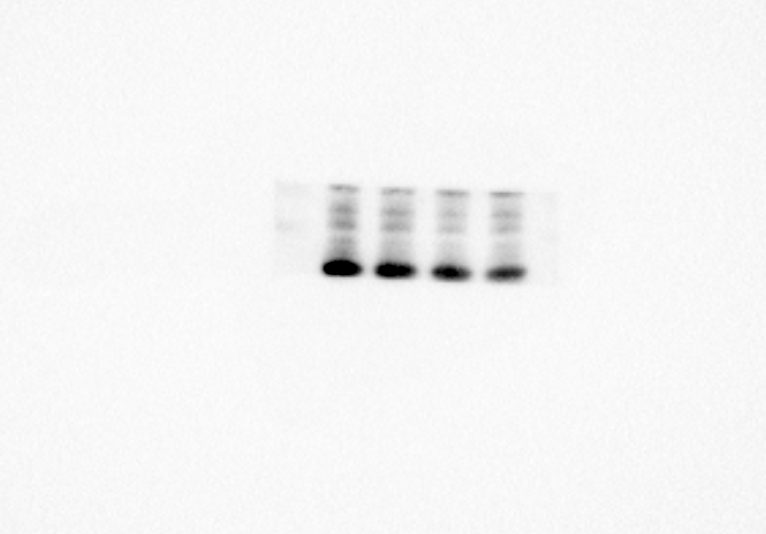

Supplement: Figure 6—source data 6. [file elife-101731-fig6-data6.zip › Figure 6-source data 6/VEGF.tif]

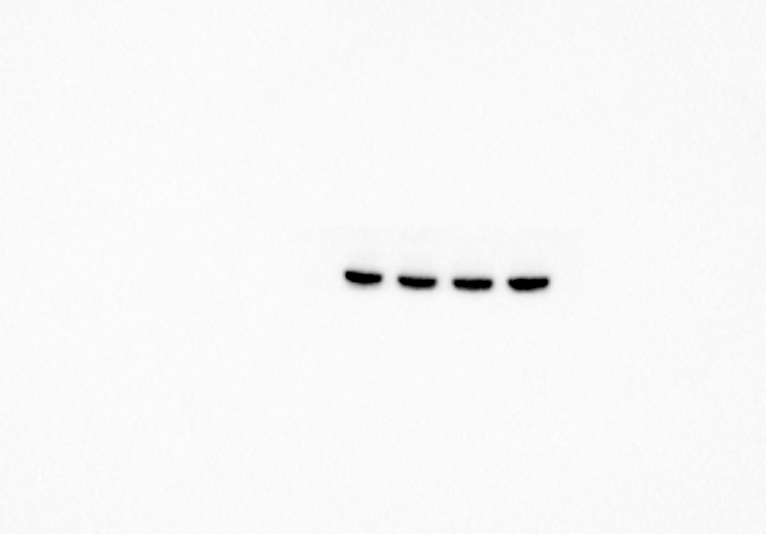

Supplement: Figure 6—source data 6. [file elife-101731-fig6-data6.zip › Figure 6-source data 6/β-Actin.tif]

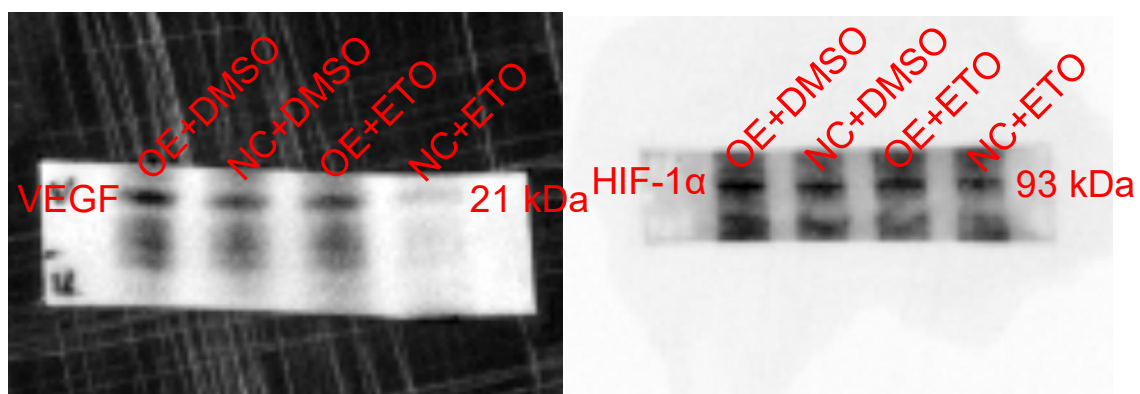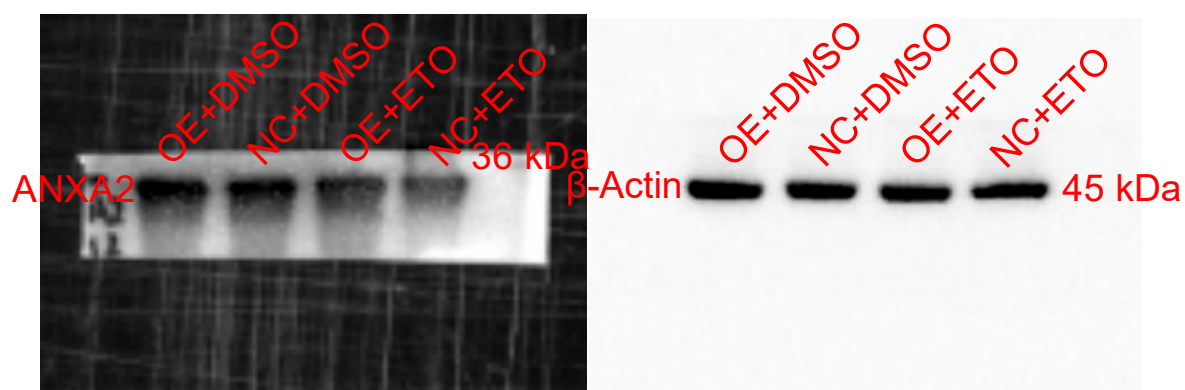

Supplement: Figure 6—source data 7. [file elife-101731-fig6-data7.zip › Figure 6-source data 7.pdf]

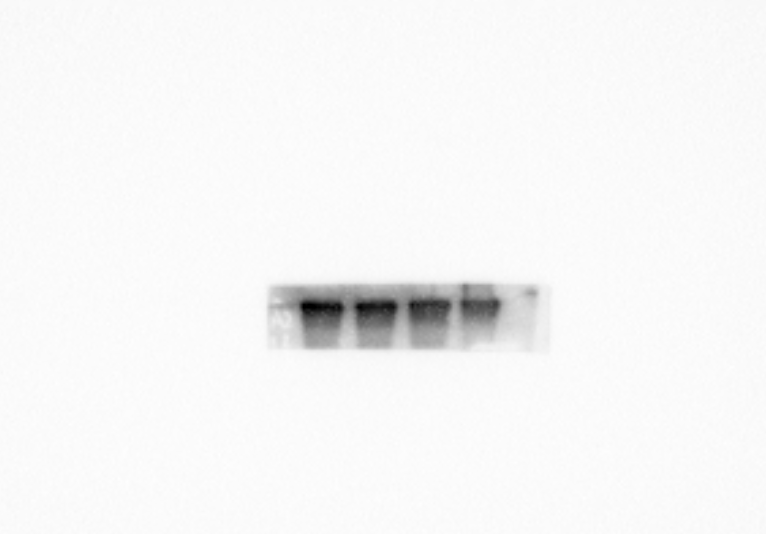

Supplement: Figure 6—source data 8. [file elife-101731-fig6-data8.zip › Figure 6-source data 8/ANXA2.tif]

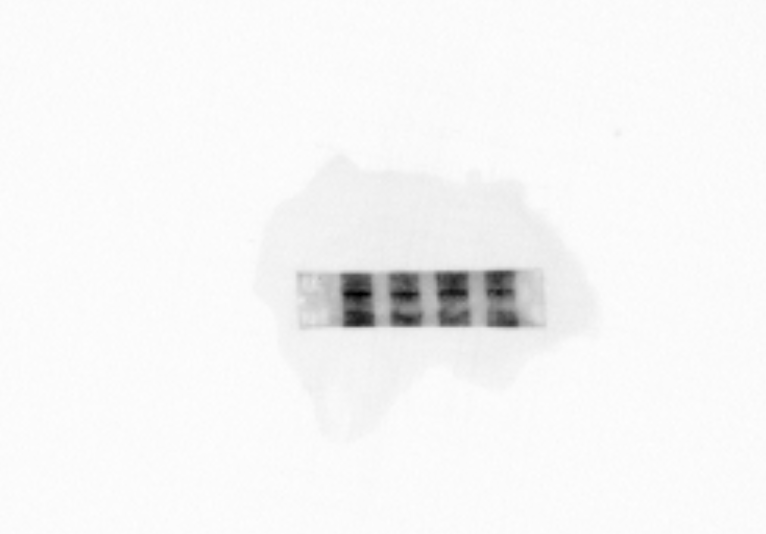

Supplement: Figure 6—source data 8. [file elife-101731-fig6-data8.zip › Figure 6-source data 8/HIF-1α.tif]

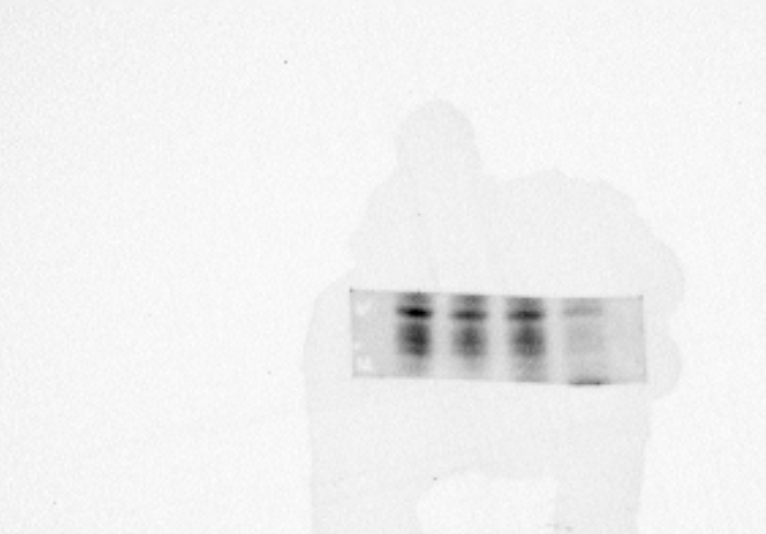

Supplement: Figure 6—source data 8. [file elife-101731-fig6-data8.zip › Figure 6-source data 8/VEGF.tif]

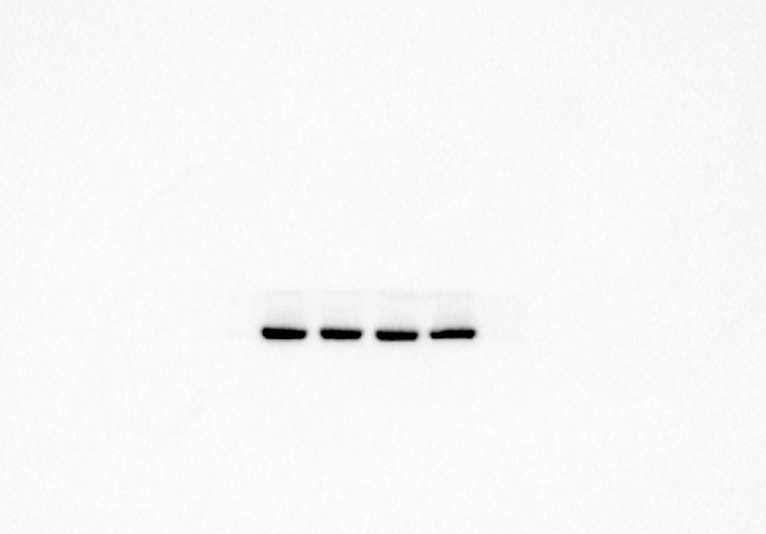

Supplement: Figure 6—source data 8. [file elife-101731-fig6-data8.zip › Figure 6-source data 8/β-Actin.tif]
